# Factors Affecting COVID-19 Preventive Behaviors among University Students in Beijing, China: An Empirical Study Based on the Extended Theory of Planned Behavior

**DOI:** 10.3390/ijerph18137009

**Published:** 2021-06-30

**Authors:** Jiabin Li, Xianwei Liu, Yang Zou, Yichu Deng, Meng Zhang, Miaomiao Yu, Dongjiao Wu, Hao Zheng, Xinliang Zhao

**Affiliations:** 1 Advising Center for Student Development, Beijing University of Technology, Beijing 100124, China; lijiabin@bjut.edu.cn; 2 Institute of Higher Education, Research Centre for Capital Engineering Education Development, Beijing University of Technology, Beijing 100124, China; 3 College of Business Administration, Capital University of Economics and Business, Beijing 100070, China; zouyang@cueb.edu.cn; 4 Publicity Department, Beijing University of Technology, Beijing 100124, China; dengyichu@bjut.edu.cn; 5 School of Civil and Transportation Engineering, Beijing University of Civil Engineering and Architecture, Beijing 102616, China; zhangmeng1@bucea.edu.cn; 6 Institute of Education Economics and Management, University of Science and Technology Beijing, Beijing 100083, China; yumiaomiao@ustb.edu.cn; 7 School of Marxism, Beihang University, Beijing 100191, China; wudongjiao@buaa.edu.cn; 8 China Youth & Children Research Center, Beijing 100089, China; zhenghao@buaa.edu.cn; 9 Beijing Academy of Educational Sciences, Beijing 100045, China; xlzhao@ciefr.pku.edu.cn

**Keywords:** institutional climate, COVID-19 preventive behaviors, extended theory of planned behavior, university students

## Abstract

Higher education institutions (HEIs), among other social systems, have an irreplaceable role in combating COVID-19. However, we know little about institutional and individual factors that might facilitate university students’ beliefs and behaviors toward preventive behaviors for COVID-19 within the higher education context. Our study applies an extended theory of planned behavior (TPB) model to investigate the structural relationships among the institutional climate, attitudes, subjective norms, perceived behavioral control and preventive behaviors of university students and to detect the moderating impacts of perceived risk on the structural model. Data were collected from 3693 university students at 18 universities in Beijing, China through an online survey. Structural equation modeling (SEM) and multigroup analysis were performed to examine the empirical model. The results reveal that (1) the institutional climate has a significant, direct effect on preventive behaviors for COVID-19 among university students, (2) the TPB components, namely attitudes, subjective norms and perceived behavioral control, partially mediate the relationship between the institutional climate and preventive behaviors for COVID-19, and (3) perceived risk moderates several paths in the model. Theoretical and practical implications are offered, and recommendations for future research are outlined.

## 1. Introduction

The COVID-19 pandemic swept across the world and has been deemed the most devastating disease since the Spanish Flu in 1918–1919 [1]. By the end of January 2021, COVID-19 caused over one hundred million confirmed infections and two million deaths worldwide [2]. Although the first confirmed case was identified in Wuhan in December 2019, and the disease rapidly spread to other parts of China, through strict and effective preventive regulations and fully implemented policies, China was successful in keeping the COVID-19 pandemic under control with the efforts of the entire society.

COVID-19 prevention and control posed greater challenges and more stringent requirements for higher education institutions (HEIs) than for other social organizations. In contrast to other social systems, HEIs have a high density of people, which means that once one student gets it, large-scale pandemic transmission is likely to be triggered due to the high rate of spread of COVID-19 in crowded settlements [3]. China’s health authorities responded early and quickly regarding COVID-19 prevention in HEIs. In late January 2020, soon after the outbreak of COVID-19, China’s Ministry of Education (MOE) issued a series of notices requesting that all educational institutions take effective epidemic prevention and control measures and postpone the start of the 2020 spring semester. On 13 April 2020, the MOE and the National Health Commission (NHC) released the Scheme on COVID-19 Prevention and Control in HEIs and proposed that comprehensive preventive measures be implemented before, during and after students’ return to campus. After campuses reopened, the Guidelines on COVID-19 Prevention and Control in HEIs for the 2020 autumn semester and for the 2021 spring semester were issued successively by the MOE and NHC. HEIs in China also actively displayed their major functions (talent training, scientific research and social services) during the COVID-19 pandemic by providing professional personnel, knowledge, skills and resources to combat COVID-19 and contribute to the research and development of definitive vaccines and forms of therapy.

Although some countries and regions, including China, have introduced a wide range of vaccinations, given the long-term complexity of the global pandemic situation, the construction and promotion of the preventive literature and behavior are critical in the “new normal” period of COVID-19 in addition to treatment and vaccine development. In fact, increasing numbers of studies added to the understanding of general public preventive behavior in many countries after the outbreak of COVID-19 [4,5,6,7,8,9]. However, few have targeted higher education systems [10,11], and most have focused on the role of knowledge and attitudes in predicting students’ COVID-19 preventive behavior [12]. Thus, although HEIs are regarded as effective settings to shape specific attitudes and behaviors of students through institutional interventions [13], we still have limited knowledge about how the institutional factors of HEIs are affecting the preventive behaviors of university students and the psychological mechanism underlying this relationship during the outbreak of the COVID-19 pandemic.

To overcome the limitations of existing studies, our study explicitly identifies the impacts of the institutional climate, attitudes, subjective norms, perceived behavioral control and perceived risk of university students’ COVID-19 preventive behaviors based on an extended theory of planned behavior (TPB) model. The TPB model proposed by Ajzen [14] may be one of the most influential theoretical perspectives to explain a range of health behavior intentions or actual behavior. While the TPB has been used in several recent studies of COVID-19 preventive behaviors [15], to improve the predictive capabilities of the TPB model, it is necessary to study university students’ COVID-19 preventive behavior by including the institutional climate in the TPB model, because the critical role of HEIs in the prevention and control of COVID-19 has been widely reported in the literature [16]. Although the existing literature recognizes the moderating role of risk perception on a range of health behaviors [17], to the best of our knowledge, no previous study has examined the variations in the influence of institutional factors and TPB components on COVID-19 preventive behaviors in terms of different levels of risk perception.

Bearing the above considerations in mind, the key objective of the current research is to employ an extended TPB model to (1) explore the influence of the institutional climate on the COVID-19 preventive behaviors of university students, (2) test the mediating effect of three TPB elements, namely attitudes, subjective norms and perceived behavioral control toward COVID-19 prevention in the relationship between the institutional climate and university students’ preventive behaviors, and (3) investigate the moderating role of perceived risk for the impacts of the institutional climate and TPB elements on university students’ preventive behaviors. The results of our study will contribute to widening the reach of the application of the TPB model in COVID-19 prevention within a higher education context and improve its explanatory capacity by adding external institutional factors and internal perceived risk. Notably, with deeper knowledge of the drivers of university students’ preventive behaviors, the present research can shed further light on anti-epidemic practices and measures in HEIs in China and in other areas suffering the devastating effects of COVID-19.

## 2. Theoretical Framework and Hypotheses

### 2.1. Extended TPB and Preventive Behaviors for COVID-19

Ajzen [14] introduced the TPB model as an extension and improvement of the theory of reasoned action [18]. The TPB model consists of three exogenous constructs, namely attitudes, subjective norms and perceived behavioral control. The underlying premise of the TPB model is that individuals’ attitudes toward behavior, subjective norms and perceived behavioral control together can shape their behavioral intentions or actual behaviors. Since its proposal, the TPB model has been extensively used to understand behaviors in a variety of domains, including a number of health-related behaviors [19,20,21]. Recent studies have also provided empirical support for the utility of the TPB model in explaining COVID-19 preventive behaviors. For instance, Prasetyo et al. [22] assessed the variables that affect the perceived effectiveness of COVID-19 prevention measures in the Philippines based on TPB and protection motivation theory. Sturman et al. [23] established a modified TPB model by incorporating knowledge to better understand adherence to restrictions during the COVID-19 pandemic by respondents in metropolitan Melbourne, Australia. Furthermore, Trifiletti et al. [24] used the TPB model along with perceived risk to evaluate protective behavior against COVID-19 in adults residing in Italy. The studies mentioned above indicate that the TPB model may benefit from reasonable expansion or modification to make it suitable for preventive behaviors for COVID-19 within different contexts.

**Figure 1:**
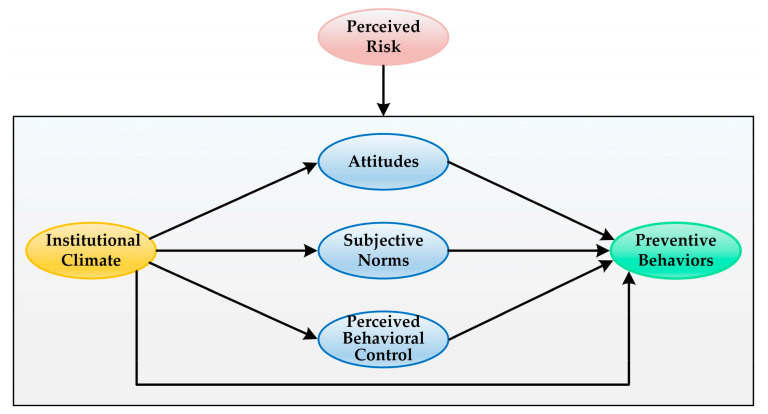
Research model.

The literature in the higher education research field has explicitly elaborated the critical impact of institutional factors, such as the structures, policies and practices of HEIs, on students’ experiences, beliefs, attitudes and behavioral outcomes [25,26]. After systematically comparing the TPB model and several other behavioral theories applied in the research of infection control practices, Kretzer and Larson [27] noted that real behavioral change does not happen by targeting the individual alone; the institutional context must also be taken into consideration when seeking to improve infection control practices. Based on these perspectives, our study was designed to include institutional climate in rgw TPB model as an antecedent factor of both TPB variables and preventive behaviors for COVID-19. In addition, COVID-19 has been proposed to be more dangerous and unpredictable than other infectious diseases [28], and different students may possess different levels of risk in the same environment. Therefore, we further considered perceived risk associated with COVID-19 as a moderating factor in the extended TPB model. The extended TPB model in the current study is presented in Figure 1.

### 2.2. Institutional Climate and Preventive Behaviors for COVID-19

Institutional climate is defined here as university students’ perception of their affiliated HEI’s formal policies, procedures and practices concerning the prevention and control of COVID-19 on campus [29]. According to previous research, organizational factors within the public sector play a vital role in the prevention and control of epidemics [30]. Researchers have also provided abundant empirical evidence for the relationship between institutional factors and various kinds of individuals’ health-related behaviors. For example, Cheung [31] found that organizational regulation of worksite noise helped promote employees’ protective behaviors for hearing loss. Ko and Kang [32] revealed that the organizational climate had a positive and significant influence on school dietitians’ food safety and hygiene behaviors. Schwatka et al. [33] found that organizational safety and the health climate were positively correlated with the healthy behaviors of workers in small businesses. During the COVID-19 pandemic, although no study has directly examined the relationship between the institutional climate and preventive behaviors of COVID-19, several recent studies have provided insight into the role of institutional factors that protect against the negative effects of the COVID-19 pandemic and promote precautionary actions to minimize risk while sustaining psychological wellbeing [34,35]. In particular, Tausen et al. [36] reported that the response and support of universities combating COVID-19 increased the subjective well-being of Asian students at a predominantly white university during the COVID-19 crisis in the US. Thus, based on previous evidence, our study proposes the following hypothesis:

**Hypothesis** **1** **(H1).** *The institutional climate is positively associated with university students’ preventive behaviors for COVID-19.*

### 2.3. Mediating Role of TPB Components

The mediation effect refers to the effect of an independent variable on a dependent variable transmitted through an intervening variable [37]. Following the definition given by Ajzen [14], attitudes refer to university students’ positive or negative feelings toward and evaluations of actions against COVID-19. Subjective norms refer to university students’ perceptions of social pressure to adopt or not adopt COVID-19 preventive behavior. Perceived behavioral control refers to university students’ perceived ease or difficulty of adopting preventive behaviors for COVID-19. Based on the perspective of the TPB model, the more favorable students’ attitudes and subjective norms are with respect to preventive behavior for COVID-19, and the greater the perceived behavioral control is, the more likely students are to perform preventive behaviors [14]. The recent literature has demonstrated the significance of the three TPB components in predicting a series of preventive behaviors for COVID-19. For example, Duong et al. [38] found that citizens’ attitudes exerted a positive and significant effect on both social distancing behavior and mask wearing behavior in the U.S. Chen and Chen [39] found that attitudes and subjective norms had a significant and positive influence on individuals’ preventive behaviors for COVID-19 among 1591 residents in China. Prasetyo et al. [22] revealed the positive impacts of the three TPB variables on the intention to follow preventive measures for COVID-19 in a sample in the Philippines.

Scholars have pointed out that the organizational and contextual factors connected to the formation of TPB components are not clearly represented [40]. A few studies have shed light on the understanding of the institutional factors associated with TPB components in the context of disease prevention. For example, an empirical study by Siuki et al. [41] revealed that health education interventions regarding HIV and AIDS prevention behaviors exerted a significant impact on the attitudes, subjective norms and perceived behavioral control among health volunteers in Iran. Lee and Li [42] demonstrated that organizational trust was related to individuals’ perceived norms and perceived behavioral control toward social distancing behavior during the early stages of COVID-19 in the United States. Based on the extended TPB model and existing empirical evidence, we argue that the institutional climate may first influence TPB components and then enhance students’ preventive behaviors for COVID-19. Thus, the following hypotheses are proposed:

**Hypothesis** **2a** **(H2a)**. *Attitudes mediate the relationship between the institutional climate and preventive behaviors for COVID-19.*

**Hypothesis** **2b** **(H2b)**. *Subjective norms mediate the relationship between the institutional climate and preventive behaviors for COVID-19.*

**Hypothesis** **2c** **(H2c)**. *Perceived behavioral control mediates the relationship between the institutional climate and preventive behaviors for COVID-19.*

### 2.4. Moderating Role of Perceived Risk

The moderating effect refers to the direction or strength of the relationship between two variables being influenced by a third variable [43]. Perceived risk is defined as one’s psychological judgments and subjective feelings concerning the consequences and probability of an adverse event such as a pandemic [44]. Generally, perceived risk is a critical antecedent of individuals’ health protective behaviors in empirical studies [45], whereas little is known about its possible moderating effect on specific relationships despite recent attention. For instance, Roma et al. [46] demonstrated that perceived risk can moderate the effect of the perceived efficacy of government guidelines on compliance with COVID-19 protective measures, as well as the impact of perceived efficacy on self-efficacy and the influence of self-efficacy on compliance. Consistent with these findings, we propose that the performance of preventive behaviors by university students might vary according to the level of the students’ perceived risk. In other words, university students with different risk perceptions who are exposed to similar institutional climates on their campus may nonetheless engage in different preventive behaviors due to differences in how they evaluate the probability and severity of COVID-19 infection. Hence, the present study aims to test the impact of perceived risk on the link between the institutional climate and preventive behaviors with TPB components as mediating variables. Accordingly, the following hypothesis is suggested:

**Hypothesis** **3** **(H3)**: *The perceived risk moderates the relationships among the institutional climate, TPB components and preventive behaviors for COVID-19.*

## 3. Methodology

### 3.1. Sample and Data Collection

Our study targeted university students in Beijing because it is the capital city and educational center of China and one of the most populous cities in the world. We used the 2021 Higher Education and Sustainability Survey (HESS) and its COVID-19-specific module. The HESS employed a random sampling design to guarantee that the resulting sample was representative of college students in Beijing. During the epidemic period, with the assistance of the student activity directors or advisors of each targeted college or department, survey questionnaires were sent to 4000 university students in 18 universities via the online survey platform Wenjuanxing (https://www.wjx.cn/ accessed date: 4 January 2021) in January and February 2021. The instruction page of the survey presented the participants with the goals of the study as well as the voluntary nature, confidentiality of participation and other matters that required attention when completing the questionnaire items. A total of 3987 questionnaires were returned. We excluded questionnaires completed in less than 3 min or with 10 consecutive identical answers to ensure that all items were clearly understood by the participants. After removing 294 invalid responses, 3693 qualified questionnaires were obtained for data analysis. Table 1 summarizes the composition of the final sample.

### 3.2. Measures

The questionnaire included two parts: the background information of the respondents and measurement items of the constructs in the extended TPB framework. All of the scales were drawn from existing research or official documents, and a five-point Likert format was adopted for each item.

For the institutional climate toward COVID-19 prevention on campus, a six-item scale was adapted from the Guidelines on COVID-19 Prevention and Control in Higher Education Institutes recommended by the National Health Commission and Ministry of Education of China [47]. The respondents were asked about the extent to which they agreed with statements regarding the policies, procedures and practices against COVID-19 adopted by their respective universities (1 = strongly disagree, up to 5 = strongly agree).

Three items of the attitudes toward COVID-19-preventive behaviors were revised from Cheng and Ng [48] to assess the tendency of students to see the performance of COVID-19 preventive behaviors as benefits or barriers (1 = strongly disagree, up to 5 = strongly agree).

Three items derived from Sumaedi et al. [49] were utilized to evaluate respondents’ subjective norms, namely the perception of social expectations from other important people to engage in COVID-19 preventive behaviors (1 = strongly disagree, up to 5 = strongly agree).

For perceived behavioral control, three items drawn from Prasetyo et al. [22] were used to measure the students’ perceptions of their degree of control over the adoption of COVID-19-preventive behaviors.

COVID-19 preventive behaviors were evaluated using seven items obtained by Liu et al. [50], based on the preventive measures officially recommended by the Chinese Center for Disease Control and Prevention. We asked respondents how often they had adopted seven different COVID-19 preventive behaviors during the epidemic period (1 = never, up to 5 = always).

For the moderator, three items of the perceived risk scale were adapted from Ma [51] to measure the respondents’ judgments concerning the adverse outcomes of COVID-19. Moreover, to examine the moderating role of perceived risk in the hypothesized path model, we used the median split approach to divide the sample into two subgroups of high and low risk perception students (Md = 3.33). The high risk perception group consisted of 1270 respondents, and the low risk perception group consisted of 1816 respondents. For more precise analysis, we omitted the data from respondents on the median (*n* = 607). We coded this as a dummy variable in the data analyses (0 = low perceived risk, 1 = high perceived risk).

As Table 2 illustrates, the Cronbach’s α coefficients of the six scales ranged from 0.710 to 0.942, greater than the threshold level of 0.700 [52]. The mean score of the items ranged from 3.915 to 4.528, the standard deviation varied from 0.656 to 0.972, the absolute values of skewness ranged from 0.049 to 1.857 (less than 3), and the absolute value of kurtosis ranged from 0.224 to 7.209 (less than 10), suggesting that the distribution of all the variables and items was not significantly different from normality and that follow-up data analyses could be performed [53]. 

### 3.3. Data Analysis

The hypothesized relationships in the proposed model were examined through structural equation modeling (SEM) based on the maximum likelihood estimation method. The analysis adopted the two-step approach advocated by Anderson and Gerbing [54], namely measurement model evaluation followed by structural model evaluation. The indexes that detected the goodness of fit of the model included the goodness of fit index (GFI ≥ 0.90), comparative fix index (CFI ≥ 0.90), incremental fit index (IFI ≥ 0.90), Tucker–Lewis index (TLI ≥ 0.90), standardized root mean square residual (SRMR < 0.08), root mean square error of approximation (RMSEA < 0.08) and ratio of the chi-square to the degree of freedom (*χ*^2^/*df* ≤ 5). As *χ*^2^/*df* was vulnerable to the sample size, when all 3693 responses were used, the other fit indexes mentioned above may have reflected the model fit more correctly [55]. We utilized the bootstrapping procedure with 2000 bootstrap samples to obtain bias-corrected estimates of the indirect effects of the institutional climate on preventive behavior (via attitudes, subjective norms and perceived behavioral control) and their associated 95% confidence intervals (CIs). The 95% bias-corrected bootstrap CI excluded zero, suggesting a significant mediation effect. The bootstrapping method has been found to be a more accurate test of mediation effects than other available strategies such as the Sobel test, as it enabled us to prevent type I errors that might have occurred from non-normal distributions of the mediation effects [56]. Furthermore, multigroup SEM analysis was performed to investigate the moderating effect of the perceived risk, which is regarded as a more statistically effective and powerful approach to examine structural invariance [57]. All the aforementioned analyses were conducted using the Amos 23 statistical package.

## 4. Results

### 4.1. Measurement Model

**Figure 2:**
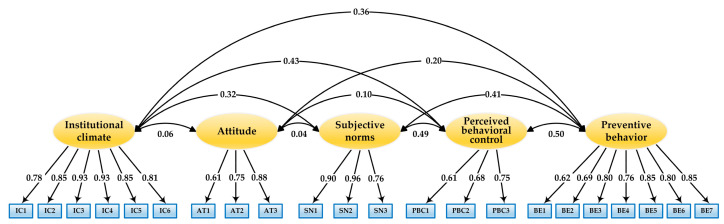
Results of the CFA.

Confirmatory factor analysis (CFA) was first conducted to confirm the fitness of the measurement model to the research data before structural model testing. The measurement model included five latent constructs and 22 observed indicators. In the CFA, we allowed the latent variables to correlate with each other, and the observed indicators were restricted to load only on their associated constructs. The CFA results showed that all the fit indexes were within acceptable ranges, except the *χ*^2^/*df* value (*χ*^2^ = 2626.013; *df* = 199; *χ*^2^/*df* = 13.196; GFI = 0.935; CFI = 0.955; IFI = 0.955; TLI = 0.947; SRMR = 0.046; and RMSEA = 0.057 (90% CI: 0.056, 0.059)). However, given the large sample size of the current study, the model fit was considered satisfactory [55]. In addition, the standardized factor loadings of all the indicators were significant and larger than the benchmark of 0.50 (from 0.609 to 0.963) [58]. Figure 2 displays the results of the measurement model.

Furthermore, we ran Harman’s one-factor test to examine the common method variance in the data [59]. We compared the fit of a single (common method) factor model with the proposed five-factor model. The results showed that the single factor model (with all the items loaded onto one latent construct) had an unsatisfactory fit to the data (*χ*^2^ = 28,647.504; *df* = 209; *χ*^2^/*df* = 137.069; GFI = 0.457; CFI = 0.469; IFI = 0.469; TLI = 0.413; SRMR = 0.186; and RMSEA = 0.192 (90% CI: 0.190, 0.194)). The chi-square statistic (Δ*χ*^2^ = 26,021.491, Δ*df* = 10, *p* < 0.001) also revealed that the measurement model provided a significantly better fit to the data than the single-factor model. Thus, common method variance was not significant in the present study.

Reliability and validity were assessed after the CFA analysis. As is presented in Table 3, the results for the composite reliability (CR) were between 0.724 and 0.944, which was higher than 0.7, indicating an acceptable level of internal consistency [60]. Additionally, the average variance extracted (AVE) scores ranged from 0.471 to 0.772 and were greater than the threshold value of 0.40, suggesting adequate convergent validity [61].

As can be seen in Table 4, all of the correlation coefficients among the variables were significant and had the anticipated sign. Specifically, the institutional climate was positively correlated to preventive behaviors (r = 0.343, *p* < 0.001). Attitudes (r = 0.192, *p* < 0.001), subjective norms (r = 0.405, *p* < 0.001) and perceived behavior control (r = 0.407, *p* < 0.001) were each significantly associated with preventive behaviors. The institutional climate was also significantly correlated with attitudes (r = 0.055, *p* < 0.01), subjective norms (r = 0.317, *p* < 0.001) and perceived behavior control (r = 0.352, *p* < 0.001). These correlations met the conditions for mediation suggested by Baron and Kenny [62]. Moreover, as the square roots of the AVEs for all of the constructs were higher than the correlations among them, the discriminant validity of the measurement was confirmed [61].

### 4.2. Structural Model

**Figure 3:**
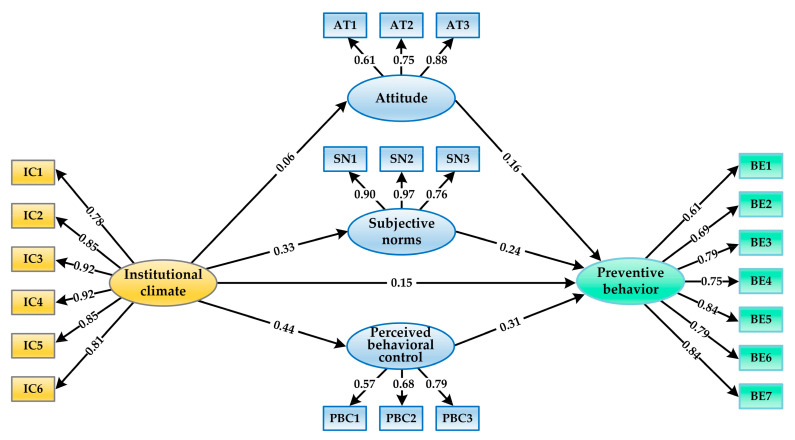
Results of the SEM.

SEM analysis was employed to evaluate the hypothesized paths in the structural model. The analysis revealed an acceptable fit of the proposed structural model to the data (*χ*^2^ = 3065.544; *df* = 202; *χ*^2^/*df* = 15.176; GFI = 0.928; CFI = 0.946; IFI = 0.947; TLI = 0.939; SRMR = 0.069; and RMSEA = 0.062 (90% CI: 0.060, 0.064)). Then, the statistical significance of the path coefficients among the constructs was estimated. As is demonstrated in Figure 3, all the direct paths were statistically significant. First, the institutional climate had significant effects on the attitudes (*β* = 0.057, *t* = 3.066, *p* < 0.01), subjective norms (*β* = 0.329, *t* = 19.026, *p* < 0.001) and perceived behavioral control (*β* = 0.437, *t* = 19.417, *p* < 0.001). Second, the direct effect of the institutional climate on preventive behaviors was significant (*β* = 0.148, *t* = 7.980, *p* < 0.01). Third, the attitudes (*β* = 0.163, *t* = 9.521, *p* < 0.001), subjective norms (*β* = 0.243, *t* = 13.351, *p* < 0.001) and perceived behavioral control (*β* = 0.308, *t* = 13.158, *p* < 0.001) exerted significant impacts on preventive behaviors. 

We ran a bootstrapping analysis to further verify the mediation effects in the hypothesized model. As is revealed in Table 5, both the direct and indirect effects of the institutional climate on preventive behaviors were significant (all 95% bias-corrected CI did not include 0), suggesting that the link between the institutional climate and preventive behaviors was partially mediated by attitudes, subjective norms and perceived behavioral control. The results indicated that university students with high perception of the institutional climate tended to express more favorable attitudes, stronger subjective norms and greater perceived behavioral control toward COVID-19 prevention, which could promote the development and performance of preventive behavior. Thus, H1, H2a, H2b and H2c were supported.

### 4.3. Moderating Effects

Multigroup SEM analyses were employed to examine the moderating effects of perceived risk in the structural model. The sample was divided into two subgroups of high and low risk perception students using the median split approach. Next, we conducted a chi-square difference test to compare a constrained model (all the paths were restricted across the two subgroups) with an unconstrained model (all the paths were not constrained across the two subgroups). If the constrained model presented a significantly larger chi-square value than the constrained model, then this implied a potential moderating effect [60]. In each model, factor loadings between the two groups were held equivalent to ensure that the variables were measured similarly across groups; however, error variances were permitted to vary between groups [63]. The chi-square statistic demonstrated that the constrained (*χ*^2^ = 3131.722, df = 428) and unconstrained models (*χ*^2^ = 3037.387, *df* = 421) were significantly different (Δ*χ*^2^ = 94.335, *df* = 7, *p* < 0.001), supporting the moderation effect of perceived risk on structural relationships.

To accurately detect the moderating effects of perceived risk on specific paths in the proposed model, a battery of chi-square difference tests was applied to compare the constrained models with seven diverse models separately, each retaining only one of the structural paths to be freely estimated. As is illustrated in Table 6, perceived risk significantly moderated four of the seven structural relationships. Specifically, the effect of the institutional climate on preventive behaviors was stronger for high risk perception students (*β* = 0.251, *t* = 8.594, *p* < 0.001) than for low risk perception students (*β* = 0.093, *t* = 3.831, *p* < 0.001). The effect of the institutional climate on subjective norms was stronger for high risk perception students (*β* = 0.379, *t* = 13.115, *p* < 0.001) than for low risk perception students (*β* = 0.292, *t* = 12.129, *p* < 0.001). The influence of the institutional climate on perceived behavioral control was significantly stronger among high risk perception students (*β* = 0.528, *t* = 15.177, *p* < 0.001) than among low risk perception students (*β* = 0.376, *t* = 13.045, *p* < 0.001). Moreover, high risk perception students (*β* = 0.281, *t* = 10.011, *p* < 0.001) exhibited a larger path effect than low risk perception students (*β* = 0.200, *t* = 8.159, *p* < 0.001) in the influence of subjective norms on preventive behavior. However, the results did not suggest the existence of significant differences between high and low risk perception groups regarding the effect of the institutional climate on attitudes, as well as the effect of attitudes and perceived behavioral control on preventive behaviors. Thus, H3 was partially supported.

## 5. Discussion and Implications

The aim of the current study was to investigate the influencing factors of preventive behaviors for COVID-19 among university students in Beijing, China. With an extended TPB framework, we tested the hypothesized relationships among the institutional climate, three components of the original TPB model and preventive behaviors, as well as the moderating role of perceived risk in the structural relationships. The major research findings are summarized and discussed as follows.

Based on the extended TPB model, we found that the institutional climate was significantly associated with university students’ preventive behaviors against COVID-19. Consistent with previous studies [35], the results imply that a positive institutional setting with formal policies, procedures and practices concerning COVID-19 prevention and control could enable university students to adaptively face epidemic challenges and facilitate their preventive actions against COVID-19. In addition, countries with different strengths of social norms (or cultural tightness–looseness) were varied in their effectiveness to combat COVID-19 [64]. Thus, a possible explanation for this relationship may be that an institutional climate creates social norms, duties, obligations and expectations within a specific institution that reinforce the preventive behaviors of students, especially those from tight cultures and collectivist societies such as China [48,64]. Moreover, according to the focus theory of normative conduct [65], the extent to which university students’ preventive behaviors are practiced is highly dependent on the saliency and level of HEIs’ COVID-19 prevention and control measures.

As expected, the results indicate that the institutional climate was significantly related to the three original TPB components, which in turn yielded a significant effect on preventive behaviors. The mediating effects of university students’ attitudes, subjective norms and perceived behavioral control on the relationship between the institutional climate and preventive behaviors were supported via a bootstrapping procedure. Specifically, all three TPB components partially mediated the relationship between the institutional climate and preventive behaviors. These results indicate that attitudes, subjective norms and perceived behavioral control are critical sociopsychological factors that link institutional intervention and students’ actual preventive behaviors toward COVID-19. The results suggest that with increasing emphasis on formal policies, procedures and practices concerning the prevention and control of COVID-19 on campus, university students may be expected to adopt more preventive behaviors, which requires them to possess an understanding of not only COVID-19 prevention knowledge, requirements and recommendations but also a positive emotional disposition, strong perception, substantial normative stimuli and the motivation to perform preventive behaviors; that is, the accessibility of external support, resources and information for COVID-19 prevention might lead to the enhancement of preventive behaviors by shaping the positive environment needed for university students’ active precautionary beliefs to flourish.

Multigroup SEM analyses indicated that perceived risk significantly moderated several paths in the research model. We found that the impacts of the institutional climate on both subjective norms and perceived behavioral control were significantly stronger among university students with a higher level of risk perception than among those with a low level of risk perception. Our study also demonstrated that the influences of the institutional climate and subjective norms on university students’ preventive behaviors were moderated by the perceived risk of COVID-19. Specifically, compared with students with a low level of perceived risk, those with a high level of perceived risk derived more benefits from the institutional climate in terms of the promotion or maintenance of preventive behaviors. These findings are highly similar to those of a recent study that found a moderating role of risk perception on the relationships among institutional factors, self-efficacy and compliance with prevention measures in Italian residents during the COVID-19 outbreak [46]. This may be explained by the fact that high risk perception students attempted to reduce their uncertainty and anxiety by resolving to accept preventive support, opinions or information from affiliated institutions and important figures and to enact preventive behaviors more strictly, while low risk perception students may have depended more on their own ability and judgment [66]. Moreover, our study revealed that the effect of the institutional climate on attitudes, as well as the influence of attitudes on preventive behaviors, remained invariant across the high and low risk perception groups. It can be concluded that, regardless of the level of university students’ perception of the risk related to COVID-19, a higher level of perception of the supportive institutional climate toward COVID-19 prevention stably fostered the formation of a positive attitude toward adopting preventive behavior and, in turn, resulted in increased performance of actual behaviors.

Our study has the following theoretical implications. First, it broadens the research on individuals’ preventive behaviors against COVID-19 from an institutional impact perspective with an expanded TPB model within the context of higher education. Although the institutional climate is known to be a key contextual factor for promoting individuals’ disease prevention actions, empirical evidence on the association between the institutional climate and preventive behaviors for COVID-19 is limited. We examined the direct influence of the institutional climate on the preventive behaviors of university students in Beijing, China to fill this gap in the literature. Second, to the best of our knowledge, this is the first attempt to explore quantitative evidence in the potential role of TPB core constructs for bridging the relationship between institutional factors and university students’ preventive behaviors toward COVID-19. Third, our study incorporates perceived risk as a moderator into the TPB model, thus providing more comprehensive insights into the influence mechanism of the institutional climate and TPB components on preventive behaviors. Moreover, our study verifies the scalability and versatility of the extended TPB model as a powerful theoretical basis for future studies of the COVID-19 preventive behaviors of other groups of people from diversified organizations around the world.

Regarding the practical implications, the findings of our study contribute to supporting HEIs’ vital functions in the “new normal” period of COVID-19 in China and offer meaningful information for authorities and HEIs to encourage the adoption of preventive actions among the general public and to prevent the spread of COVID-19. First, by making COVID-19 an urgent and vital political issue, institutional actors can play a powerful and effective role in shaping the social norms of epidemic prevention [67], because political engagement and social norms represent crucial factors in facilitating prosocial behavior [68]. Accordingly, HEIs could prompt the creation of an institutional climate for COVID-19 prevention via a series of institutional interventions, including establishing effective prevention and control measures and demonstrating commitment and concrete efforts to ensure the physical and mental health and safety of students and staff on campus and to maintain the normal functions of the institutions. Second, HEIs should contribute to the management and intervention of students’ positive psychological states, which will guide students in deciding which behaviors and protocols to pursue. Thus, we suggest that HEIs configure platforms to provide positive psychological interventions to students to stimulate them to enhance their knowledge, attitudes, norms and behavioral control toward COVID-19 prevention. Moreover, specific institutional interventions might be more efficient for individuals with a high level of risk perception. We propose that HEIs emphasize that more risk and crisis education is especially helpful for enhancing students’ beliefs regarding the obligations of the country, institutions and themselves to make successful efforts to defeat COVID-19.

## 6. Conclusions

Overall, the present study demonstrated that the main variables in the research model, including the institutional climate, attitudes, subjective norms, perceived behavioral control and perceived risk, played critical roles in predicting university students’ preventive behaviors against COVID-19. Thus, the TPB-based expansion model could be functionalized as an effective framework for understanding university students’ preventive behaviors on campus. Although promising, there are limitations that should be noted in subsequent research. The results of our study are limited by its generalizability to HEIs and university students in other parts of China and the world because the sample data were collected from university students in Beijing. Therefore, cross-regional and cross-country studies involving university students from a broader scope of HEIs are needed in the future to enhance the generalizability and validity of research findings or revise the framework utilized to understand the influential mechanism of contextual and psychological factors on university students’ preventive behaviors. Moreover, future studies should consider other potential mediation and moderation mechanisms of multiple cultural and psychological factors, through which HEIs can foster the preventive behaviors of university students due to the complexity and heterogeneity of COVID-19 spread and control around the world [69], thus producing valuable and creative theoretical and practical outcomes for combating COVID-19.

## Figures and Tables

**Table 1:** Composition of the final sample.

Variable	Group	Frequency (*n*)	Percentage (%)
Gender	Female	1857	50.3
	Male	1836	49.7
Major	Science and Engineering	2782	75.3
	Humanities and Social sciences	911	24.7
Grade	Freshman	1231	33.3
	Sophomore	897	24.3
	Junior	839	22.7
	Senior	726	19.7
Ethnicity	Han	3305	89.5
	Other	388	10.5

**Table 2:** Scale items and descriptive statistics.

Variables or Measurement Items	Mean	SD	Skewness	Kurtosis
**Institutional climate (IC) (Cronbach’s α = 0.942)**	4.334	0.655	−1.821	7.209
IC1: Providing sufficient epidemic prevention facilities	4.242	0.814	−1.336	2.710
IC2: Strengthening education on epidemic prevention knowledge	4.361	0.725	−1.622	4.939
IC3: Expanding online and offline learning resources	4.420	0.690	−1.857	6.711
IC4: Strengthening humanistic care and psychological counseling	4.387	0.703	−1.747	5.952
IC5: Formulating effective campus epidemic prevention regulations	4.391	0.744	−1.712	4.872
IC6: Providing timely and authoritative information about COVID-19	4.206	0.783	−1.251	2.802
**Attitudes (AT) (Cronbach’s α = 0.781)**	3.324	0.907	−0.316	−0.224
AT1: If I adopt the preventive measures, I will be less vulnerable to COVID-19 infection	3.690	1.139	−0.687	−0.403
AT2: If I adopt the preventive measures, they will cause inconvenience to me (R)	2.943	1.087	0.110	−0.922
AT3: If I adopt the preventive measures, I will become less anxious about contracting COVID-19	3.339	1.031	−0.401	−0.459
**Subjective norms (SN) (Cronbach’s α = 0.905)**	4.313	0.637	−0.698	0.720
SN1: People who are important to me think that I should perform preventive behavior	4.323	0.720	−1.151	2.288
SN2: People who have an influence in my life think that I should perform preventive behavior	4.305	0.705	−1.042	2.052
SN3: People whose opinion matters to me think that I should perform preventive behavior	4.310	0.659	−0.795	1.383
**Perceived behavior control (PBC) (Cronbach’s α = 0.720)**	4.010	0.625	−0.238	0.288
PBC1: I think preventive measures are easy to implement	4.085	0.741	−0.813	1.336
PBC2: I am confident that I can avoid being infected by COVID-19	3.971	0.845	−0.681	0.533
PBC3: I am confident that I have enough knowledge about COVID-19	3.974	0.754	−0.501	0.495
**Preventive behaviors (BE, Cronbach’s α = 0.904)**	4.500	0.529	−0.935	0.761
BE1: Minimize social activities; avoid infected areas; avoid crowded public places	4.361	0.746	−1.225	1.924
BE2: Wear a single-use medical face mask when visiting public places or taking public transport	4.699	0.548	−1.927	4.487
BE3: Keep your hands clean and wash your hands frequently; minimize contact with objects in public places	4.470	0.666	−1.102	1.103
BE4: Refrain from touching your mouth, nose, and eyes with unwashed hands; cover your mouth and nose with your elbow when sneezing or coughing	4.404	0.746	−1.265	1.726
BE5: Monitor your health conditions; comply with the campus epidemic prevention regulations	4.596	0.575	−1.275	1.801
BE6: Ensure your home is adequately ventilated	4.463	0.687	−1.220	1.553
BE7: Keep distance from others in public places to reduce unnecessary infection	4.509	0.649	−1.247	1.665
**Perceived risk (PR) (Cronbach’s α = 0.710)**	3.205	0.791	−0.049	0.238
PR1: Once I have cold symptoms, I will doubt whether I have been infected by COVID-19	2.920	1.065	0.186	−0.748
PR2: If there were confirmed cases in the same period of time in a place I visited, I would think I might be infected myself	3.706	0.918	−0.776	0.534
PR3: Once someone I have been in contact with has been diagnosed, I think it is only a matter of time before I get diagnosed myself	2.988	0.999	0.128	−0.344

Note: (R) = reversed item; SD = standard deviation.

**Table 3:** Standard factor loading of items and reliability of the scales.

Variables	Items	Loadings	CR	AVE
Institutional climate (IC)	IC1	0.779	0.944	0.738
	IC2	0.853		
	IC3	0.925		
	IC4	0.925		
	IC5	0.851		
	IC6	0.812		
Attitudes (AT)	AT1	0.609	0.794	0.568
	AT2	0.751		
	AT3	0.877		
Subjective norms (SN)	SN1	0.902	0.910	0.773
	SN2	0.963		
	SN3	0.761		
Perceived behavior control (PBC)	PBC1	0.613	0.726	0.470
	PBC2	0.683		
	PBC3	0.754		
Preventive behaviors (BE)	BE1	0.617	0.910	0.593
	BE2	0.693		
	BE3	0.800		
	BE4	0.758		
	BE5	0.849		
	BE6	0.797		
	BE7	0.847		

Note: CR = composite reliability; AVE = average variance extracted.

**Table 4:** Discriminant validity and correlation.

Variables	1	2	3	4	5
1. Institutional climate	*0.859*				
2. Attitudes	0.055 **	*0.754*			
3. Subjective norms	0.317 ***	0.054 **	*0.879*		
4. Perceived behavior control	0.352 ***	0.088 ***	0.446 ***	*0.686*	
5. Preventive behaviors	0.343 ***	0.192 ***	0.405 ***	0.407 ***	*0.770*

Note: Diagonal elements (in italics) are the square root of the average variance extracted (AVE). ** *p* < 0.01. *** *p* < 0.001.

**Table 5:** Results of bootstrapping.

Paths	Bootstrapping		95% Bias-Corrected CI	
	Effect	Boot S. E.	Boot LLCI	Boot ULCI
IC → AT	0.057 ***	0.019	0.021	0.094
IC → SN	0.329 ***	0.021	0.286	0.370
IC → PBC	0.437 ***	0.025	0.388	0.486
IC → BE	0.148 ***	0.022	0.104	0.193
AT → BE	0.163 ***	0.016	0.131	0.194
SN → BE	0.243 ***	0.023	0.199	0.287
PBC → BE	0.308 ***	0.024	0.262	0.355
IC → AT → BE	0.007 ***	0.002	0.002	0.011
IC → SN → BE	0.057 ***	0.007	0.044	0.072
IC → PBC → BE	0.096 ***	0.010	0.078	0.118

Note: IC = institutional climate; AT = attitudes; SN = subjective norms; PBC = perceived behavioral control; BE = preventive behaviors; LLCI = lower level confidence interval; ULCI = upper level confidence interval. *** *p* < 0.001.

**Table 6:** Results of the multigroup analysis.

	Standardized Coefficients		*χ*^2^ (*df*)	∆*χ*^2^ (∆*df*)
	Low-PR	High-PR		
Constrained Model	-	-	3131.722 (428)	-
IC → AT	0.082 **	0.069 *	3131.589 (427)	0.132
IC → SN	0.292 ***	0.379 ***	3116.001 (427)	15.721 ***
IC → PBC	0.376 ***	0.528 ***	3084.984 (427)	46.738 ***
IC → BE	0.093 ***	0.251 ***	3102.439 (427)	29.283 ***
AT → BE	0.175 ***	0.161 ***	3129.100 (427)	2.622
SN → BE	0.200 ***	0.281 ***	3125.790 (427)	5.932 *
PBC → BE	0.291 ***	0.350 ***	3130.303 (427)	1.419

Note: IC = institutional climate; AT = attitudes; SN = subjective norms; PBC = perceived behavioral control; BE = preventive behaviors; PR = perceived risk. * *p* < 0.05. ** *p* < 0.01. *** *p* < 0.001.

## Data Availability

The data used or analyzed during the current study are available from the corresponding author on request.
